# Biodistribution, Stability, and Blood Distribution of the Cell Penetrating Peptide Maurocalcine in Mice

**DOI:** 10.3390/ijms161126054

**Published:** 2015-11-19

**Authors:** Pascale Perret, Mitra Ahmadi, Laurent Riou, Sandrine Bacot, Julien Pecher, Cathy Poillot, Alexis Broisat, Catherine Ghezzi, Michel De Waard

**Affiliations:** 1 Grenoble Alpes University, 38041 Saint-Martin-d'Hères, France; mitra.ahmadi@ujf-grenoble.fr (M.A.); laurent.riou@ujf-grenoble.fr (L.R.); sandrine.bacot@ujf-grenoble.fr (S.B.); cathy.poillot@gmail.com (C.P.); alexis.broisat@inserm.fr (A.B.); catherine.ghezzi@ujf-grenoble.fr (C.G.); dewaardm@ujf-grenoble.fr (M.D.W.); 2 Radiopharmaceutiques Biocliniques, INSERM, UMR S1039, 38700 La Tronche, France; 3 Smartox Biotechnologies, Bâtiment Nanobio, 570 rue de la Chimie, 38400 Saint Martin d’Hères, France; julien.pecher@gmail.com; 4 Science and Therapeutics, LabEx Ion Channels, Grenoble Institute of Neuroscience, INSERM, U836, 38700 La Tronche, France

**Keywords:** maurocalcine, cell-penetrating peptide, *in vivo* biodistribution, drug delivery, blood stability

## Abstract

Maurocalcine (MCa) is the first natural cell penetrating peptide to be discovered in animal venom. In addition to the fact that it represents a potent vector for the cell penetration of structurally diverse therapeutic compounds, MCa also displays several distinguishing features that make it a potential peptide of choice for clinical and biotechnological applications. The aim of the present study was to gain new information about the properties of MCa *in vivo* in order to delineate the future potential applications of this vector. For this purpose, two analogues of this peptide with (Tyr-MCa) and without (Lin-Tyr-MCa) disulfide bridges were synthesized, radiolabeled with ^125^I, and their *in vitro* stabilities were first evaluated in mouse blood. The results indicated that ^125^I-Tyr-MCa was stable *in vitro* and that the disulfide bridges conferred a competitive advantage for the stability of peptide. Following *in vivo* injection in mice, ^125^I-Tyr-MCa targeted peripheral organs with interesting quantitative differences and the main route of peptide elimination was renal.

## 1. Introduction

Maurocalcine (MCa) has been extracted from the venom of the *Scorpio maurus palmatus* and identified as a 33-mer peptide [1]. Evidence that the coupling of a biotinylated derivative of MCa to streptavidin tagged with a fluorescent dye leads to fluorescence accumulation in a variety of cell types indicated that MCa could act as a peptide vector for the cell entrance of a cargo [2]. Additional studies indicated that glycoaminoglycans and negatively charged phospholipids represent membrane receptors of MCa [3]. At the structural level, MCa folds according to an inhibitor cysteine knot motif and contains three well-defined beta-strands [1]. The secondary structures are constrained by three disulfide bridges with a pattern of connectivity forming the unusual knot. A specificity of MCa is that it is heavily charged owing to the presence of basic amino acid residues. This property, along with the fact that MCa has the ability to induce cell penetration of a variety of cargo [4,5,6,7,8], led to the conclusion that MCa was the first identified toxin member of the large structurally-unrelated family of cell penetrating peptides (CPP). CPP are becoming increasingly popular as vectors for the cell entry of cargo that would otherwise not enter cells. As such, MCa demonstrated excellent vector properties for quantum dots, peptides, or drugs, and promising applications are envisioned in oncology [4,9,10,11,12]. Considering the potential of the natural form of MCa as a vector, we quantitatively investigated its cell penetration properties in a recent study. This was done by grafting an additional Tyr residue at the N-terminus of the peptide followed by appropriate iodination with ^125^I to provide first Tyr-MCa and next ^125^I-Tyr-MCa. The results indicated that dose-dependent accumulation of radioiodinated Tyr-MCa was observed in the nucleus and cytoplasm of rat F98 glioma cells with >24 h cellular retention [13]. While MCa is recognized as a competitive CPP due to its low concentration efficacy and ability to reach the cytoplasm, additional efforts were made to obtain MCa analogues deprived of undesirable pharmacological effects yet with preserved cell penetration properties. In this regard, the structural stringency observed for MCa binding to RyR1 is much higher than that observed for MCa cellular penetration. Hence, all strategies tested so far provided cell penetrating competent analogues that lacked RyR1 binding [7,8,14]. In essence, venomous toxins are delivered *in vivo* and are tailored to survive enough time within the blood stream of animal preys until the pharmacological potential of these molecules has been fully exploited. Two analogues of this peptide were synthesized in order to investigate the *in vivo* properties of MCa, namely Tyr-MCa that like the natural form of MCa contains three disulfide bridges according to the pattern of Cys^3^–Cys^17^, Cys^10^–Cys^21^ and Cys^16^–Cys^32^, and Lin-Tyr-MCa without disulfide bridges therefore lacking any three-dimensional structure [8]. The rationale underlying the synthesis of Lin-Tyr-MCa was that the synthesis of a peptide with multiple disulfide bridges might raise technical difficulties as compared to a linear peptide. The replacement of the six MCa internal cysteine residues by 2-Aminobutyric acid (Abu) residues results in a linear peptide lacking a secondary structure while retaining its CPP properties [8]. The synthesis of Tyr-MCa and Lin-Tyr-MCa as performed in the present study therefore allowed the evaluation of the role of MCa secondary structure on the *in vitro* and *in vivo* peptide stability. Specifically, each peptide contained an extra amino-terminal tyrosine residue for the purpose of radioiodination. The *in vitro* metabolic stability of ^125^I-labeled peptides as well as the body distribution pattern and route of elimination of ^125^I-Tyr-MCa were studied. The present study will help to delineate the *in vivo* potential of MCa as a CPP.

## 2. Results

### 2.1. Chemical Synthesis and Radiolabeling

**Figure 1:**
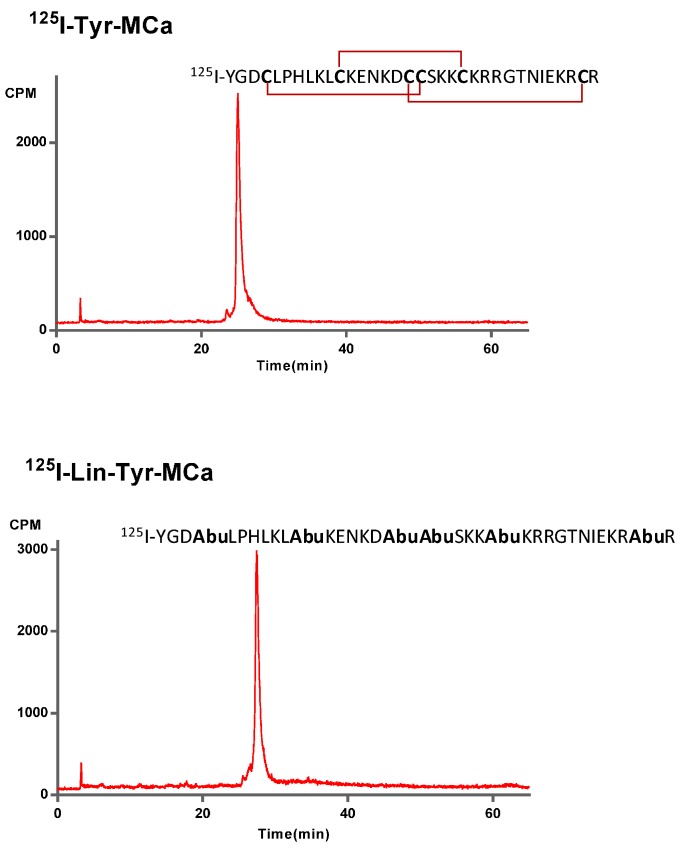
^125^I-Tyr-MCa and ^125^I-Lin-Tyr-MCa peptidic sequences and RP-HPLC profiles following radiolabeling.

MCa amino acid sequence is devoid of internal Tyr residue for peptide iodination. To facilitate the labeling of MCa analogues, we chemically synthesized the 34 amino acid Tyr-MCa and Lin-Tyr-MCa that each contains an additional Tyr residue at the N-terminus of the sequence. The Lin-Tyr-MCa contains no disulfide bridge while the Tyr-MCa folds well in spite of the extra Tyr residue and possesses the classical disulfide bridging pattern. Next, ^125^I-Tyr-MCa and ^125^I-Lin-Tyr-MCa are prepared using lactoperoxidase/H_2_O_2_ as oxidative agents. RP-HPLC analysis of the radioiodinated peptides immediately following radiolabeling is shown in Figure 1. The results indicate that the radioiodination procedure yields a single major radioactive species with radiochemical purity (RCP) >95%. ^125^I-Tyr-MCa and ^125^I-Lin-Tyr-MCa remain stable (RCP > 95%) at room temperature for at least 24 h following radiolabeling. For all subsequent experiments, ^125^I-Tyr-MCa is always used within 12 h following radiolabeling.

### 2.2. In Vitro Stability in Mouse Blood

The *in vitro* distribution pattern of radioactivity after 15, 30, 60, 90, 120 and 180 min of incubation of ^125^I-Tyr-MCa and ^125^I-Lin-Tyr-MCa with whole mouse blood is indicated in Table 1. The results indicate that the fraction of ^125^I-Tyr-MCa and ^125^I-Lin-Tyr-MCa associated to blood cells remain quite stable over time at ~20% for ^125^I-Tyr-MCa and 30% to 45% for ^125^I-Lin-Tyr-MCa. ^125^I-Tyr-MCa is mostly associated with plasma proteins (~60%–70% of total) while ^125^I-Lin-Tyr-MCa binding to plasma proteins decreases from 47% to 27% at 180 min. The protein-free plasma sample used for HPLC analysis contains ~10% of radioactivity for ^125^I-Tyr-MCa and increases progressively from 23% to 30% for ^125^I-Lin-Tyr-MCa.

**Table 1:** *In vitro* blood distribution patterns of radioactivity following ^125^I-Tyr-MCa and ^125^I-Lin-Tyr-MCa incubation with whole murine blood.

Blood Distribution	15 min	30 min	60 min	90 min	120 min	180 min
**^125^I-Tyr-MCa**						
Blood cells	22 ± 3	21 ± 3	22 ± 1	19 ± 3	18 ± 0	19 ± 2
Plasma proteins	64 ± 3	65 ± 5	64 ± 3	67 ± 3	69 ± 2	70 ± 2
Protein-free plasma	12 ± 4	12 ± 4	12 ± 3	12 ± 1	11 ± 1	9 ± 2
**^125^I-Lin-Tyr-MCa**						
Blood cells	30 ± 5	31 ± 8	35 ± 3	35 ± 9	37 ± 9	44 ± 10
Plasma proteins	47 ± 3	42 ± 3	34 ± 2	32 ± 0.4	34 ± 1	27 ± 5
Protein-free plasma	23 ± 4	23 ± 2	22 ± 3	30 ± 10	28 ± 8	28 ± 8

All of the results are expressed as %.

**Figure 2:**
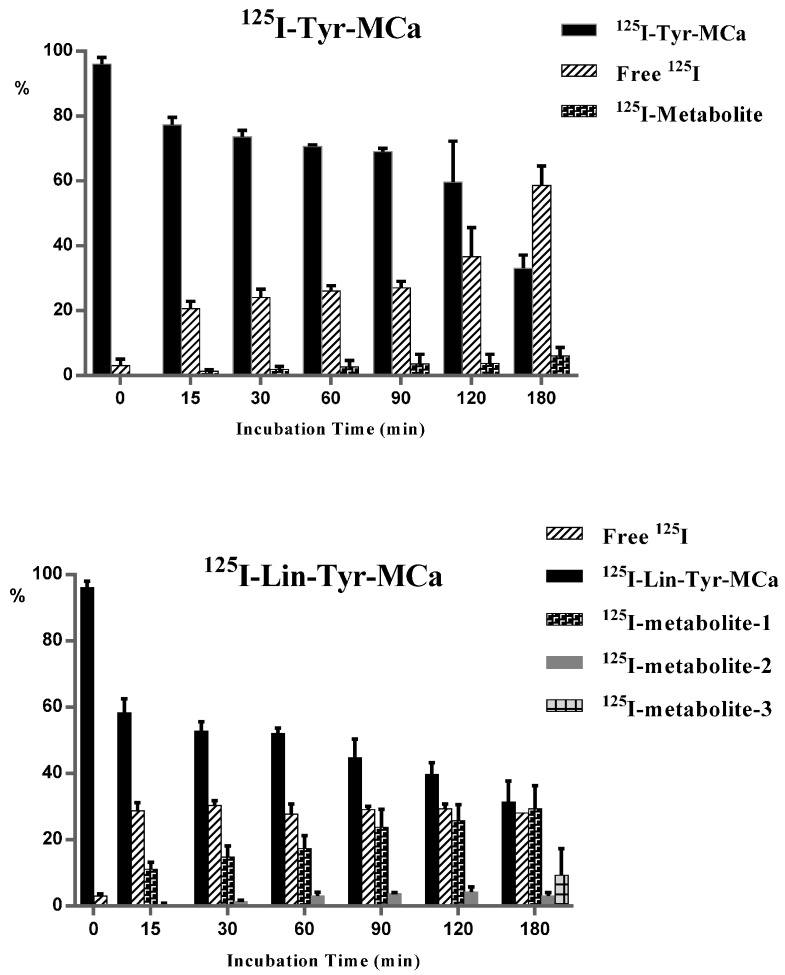
Evaluation of ^125^I-Tyr-MCa and ^125^I-Lin-Tyr-MCa stabilities following 15 to 180 min of incubation with whole murine blood. The 0 min time point refers to ratios observed immediately following radioiodination.

Radioactive species ratios calculated from HPLC analyses of the protein-free plasma fraction of mouse blood samples following 15 to 180 min of incubation with the tracers are shown in Figure 2. In this figure, the 0 min time point corresponds to radioiodinated Tyr-MCa and Lin-Tyr-MCa immediately following radiolabeling and before contact with mouse blood. The iodinated metabolite of ^125^I-Tyr-MCa represents 1.3% ± 0.5% of the total radioactivity at 15 min and increases to 6% ± 2% at 180 min, indicating that the peptide retains excellent stability. The amount of free ^125^I is stable over 90 min and represents ~20%–25% of the protein-free plasma fraction of radioactivity and then increases up to ~58% at 180 min, indicating good ^125^I-Tyr-MCa stability for 90 min. Single-photon emission computed tomography (SPECT) imaging and biodistribution studies are performed up to 60 min following injection in order to account for the increased deiodination observed thereafter. In this setting, the ~20%–25% of free ^125^I detected in the protein-free plasma fraction of radioactivity correspond to ~4% of total blood activity since free ^125^I is exclusively located in the protein-free plasma fraction as shown by results from TLC analysis (data not shown).

^125^I-Lin-Tyr-MCa is degraded as early as 15 min of incubation with appearance of 11% ± 2% of a first iodinated metabolite (^125^I-metabolite-1). A second iodinated metabolite (^125^I-metabolite-2) appears after 15 min of incubation which proportion increases to 4.3% ± 1% at 120 min. Finally, a third iodinated metabolite (^125^I-metabolite-3) appears at 180 min while the presence of ^125^I-metabolite-1 and ^125^I-metabolite-2 is concomitantly observed. The amount of free ^125^I is stable (~30%) and ^125^I-Lin-Tyr-MCa decreases to 31.3% ± 6% after 180 min of incubation.

Radioiodinated metabolites of ^125^I-Lin-Tyr-MCa evidenced by *in vitro* experiments can interfere *in vivo* biodistribution and imaging studies. Accordingly, all subsequent *in vivo* tracer evaluations are performed using ^125^I-Tyr-MCa only.

### 2.3. In Vivo Tracer Stability

*In vivo* blood distribution pattern of radioactivity following ^125^I-Tyr-MCa intravenous injection is indicated in Table 2. As observed *in vitro*, the radioactivity is mostly associated with plasma proteins, although to a slightly lower level. The remaining activity is equally distributed between blood cells and the protein-free plasma fraction. HPLC analysis of radioactive species in the protein-free plasma fraction of mouse blood at 15 and 30 min post-injection in mice pretreated by the NaI symporter inhibitor potassium perchlorate indicates the presence of free ^125^I and ^125^I-Tyr-MCa. Free ^125^I represents ~62% of the total protein-free plasma radioactivity at 15 min, a value which increases slowly (~72% at 30 min). Considering that the protein-free plasma fraction of blood contains ~29% of the total blood activity (Table 2), one can therefore estimate that free ^125^I represents ~18% of the total circulating activity following *in vivo* injection of the tracer. Rather than indicating an increased deiodination of the tracer following *in vivo* injection, the apparently higher proportion of free ^125^I following *in vivo* injection of ^125^I-Tyr-MCa (~18% of total blood activity) as compared with results from *in vitro* incubation of the tracer with mouse blood (~4% of total blood activity) is likely due to the fact that unbound ^125^I-Tyr-MCa is available for organ distribution following *in vivo* injection in mice, whereas this is not the case following *in vitro* blood incubation of the tracer. These observations indicate that ^125^I-Tyr-MCa is probably accumulating in cells of various organs as expected for a cell penetrating peptide.

**Table 2:** *In vivo* blood distribution pattern and stability analysis of ^125^I-Tyr-MCa in protein-free plasma at 15 and 30 min following potassium perchlorate pretreatment and intravenous injection to mice.

	Blood Distribution		15 min	30 min
^125^I-Tyr-MCa	Blood cells		24	31
	Plasma proteins		47	42
	Protein-free plasma		29	27
		Free ^125^I	62	72
		^125^I-Tyr-MCa	38	28

All of the results are expressed as %.

### 2.4. Biodistribution

**Figure 3:**
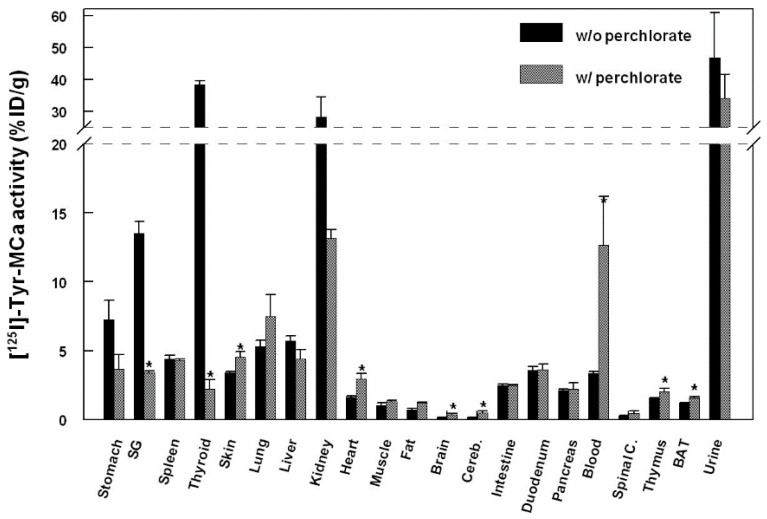
Biodistribution of ^125^I-Tyr-MCa in CD-1 mice at 60 min post-injection and effect of the NaI symporter inhibitor potassium perchlorate. SG, salivary gland; Cereb., cerebellum; Spinal C., Spinal Cord, BAT, Brown Adipose Tissue. * *p* ≤ 0.05.

The biodistribution of ^125^I-Tyr-MCa in CD-1 mice at 60 min following tracer injection is shown in Figure 3. The tracer is mainly eliminated through the kidneys as shown by the high renal and urinary activities. High activities are also observed in the stomach, salivary gland, and thyroid, which correspond to ^125^I uptake by these organs. ^125^I-Tyr-MCa does not cross the blood brain barrier, or at least badly, as shown by the extremely low brain and cerebellum activities. Figure 3 also compares the biodistributions of ^125^I-Tyr-MCa with a pretreatment with the NaI symporter inhibitor, potassium perchlorate, to the condition without this pretreatment. As expected, the presence of potassium perchlorate significantly inhibits ^125^I uptake by the salivary glands and thyroid, which likely accounts for a significantly increased amount of blood circulating radioactivity. The biodistribution patterns are otherwise comparable between the two experiments.

### 2.5. In Vivo Imaging

**Figure 4:**
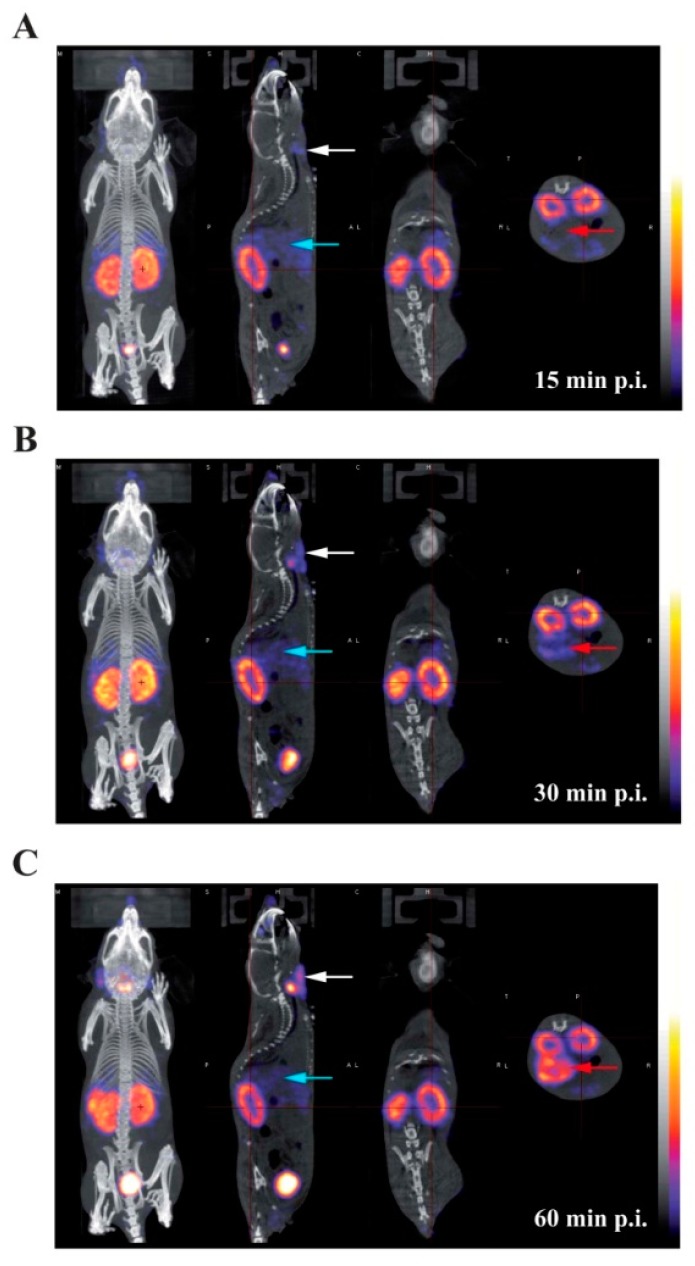
Whole-body SPECT/CT *in vivo* imaging of ^125^I-Tyr-MCa in CD-1 mice at 15 min post-injection (**A**); 30 min post-injection (**B**); and 60 min post-injection (**C**); from left to right, 3D rendering, sagittal, coronal, and transverse views of tracer activity. White, blue and red arrows are respectively pointing at salivary gland, liver, and stomach activity.

**Figure 5:**
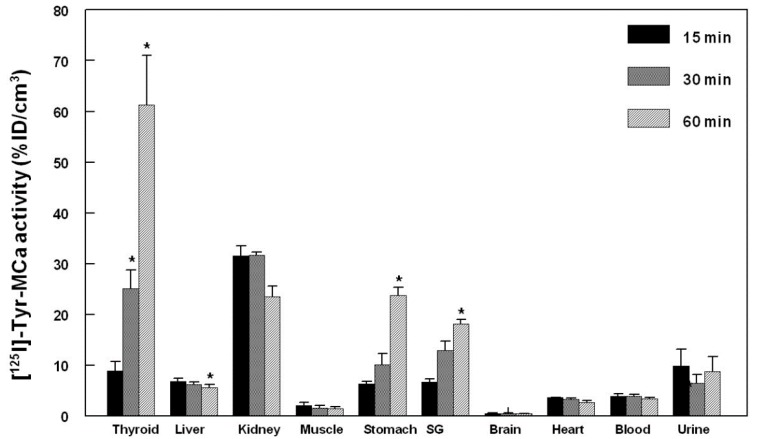
Quantification of *in vivo* tomographic images of ^125^I-Tyr-MCa whole-body distribution at 15, 30, and 60 min post-injection. * *p* ≤ 0.05 *versus* 15 min.

Presented in Figure 4 are representative images acquired in the same animal at 15 min (panel A), 30 min (panel B), and 60 min (panel C) following the intravenous injection of ^125^I-Tyr-MCa. The results from *in vivo* image quantification are presented in Figure 5. Non-invasive 60-min image quantification accurately reflects *post-mortem* organ biodistribution. As shown on images and as confirmed following image quantification, there is a progressive accumulation of ^125^I in the thyroid, stomach, and salivary glands from 0 to 60 min post-injection. Renal elimination of the tracer begins immediately following injection and remains stable over time. Hepatic elimination of ^125^I-Tyr-MCa is lower than that occurring through the kidneys and is also stable over time.

## 3. Discussion

MCa has *potential* as a cell penetrating peptide for drug delivery [11]. A better understanding of the *in vivo* behaviour of MCa following systemic delivery is therefore an important step in the development of the peptide. Accordingly, the present study reports the *in vitro* and *in vivo* stability as well as the *in vivo* biodistribution of radiolabeled MCa following intravenous injection in mice.

The role of the secondary structures on MCa stability and biodistribution was investigated by synthesizing Lin-Tyr-MCa containing no disulfide bridge and Tyr-MCa containing three disulfide bridges as encountered in the natural form of the toxin. The initial rationale for using Lin-Tyr-MCa lied in the lower technical difficulty related to the production of a peptide without multiple disulfide bonds. Both peptides were then successfully radioiodinated according to previously described [15] with excellent radiochemical stability over 24 h. The *in vitro* stability of both radiolabeled peptides was then evaluated in mouse blood. Lin-Tyr-MCa was degraded as early as 15 min following initial contact with mouse blood leading to the appearance of two additional radiolabeled products demonstrating the instability of ^125^I-Lin-Tyr-MCa most likely due to the absence of disulfide bridges. Because of such degradation, the *in vivo* evaluations performed in the present study used the relatively natural form of Tyr-MCa that has shown an excellent stability in the presence of mouse blood *in vitro*.

The *in vivo* ^125^I-Tyr-MCa biodistribution experiments reported in the present study were performed at blood peptide concentrations in the range of 1 µM which are therefore representative of the effective concentrations required for cell penetration and pharmacological action [5].

The present study led to several interesting conclusions. First, Tyr-MCa displayed blood stability in our experimental conditions. Apart from modest deiodination yielding free ^125^I (see below), the peptide showed little degradation in agreement with the expected stability of disulfide-bridged toxins *in vivo* [16,17]. The three disulfide bridges that connect the six internal cysteine residues of MCa may confer a relatively high resistance to proteases. This finding further reinforces the competitiveness of MCa as a cell delivery vector. Second, the present study allowed important conclusions to be drawn regarding the fate of the peptide following intravenous injection. Both *in vitro* and *in vivo* data provide consistent conclusions. Up to 22% of ^125^I-Tyr-MCa was associated with blood cells, which is a first indication that ^125^I-Tyr-MCa has the ability to enter these cell types. The type of blood cell being preferentially targeted by ^125^I-Tyr-MCa remains to be determined, should MCa be used for targeted blood cell delivery. In addition, the observed values of thyroid activity were consistent with modest *in vivo* deiodination as previously demonstrated by Huang *et al.* [18]. Third, the data clearly demonstrated that ^125^I-Tyr-MCa did not reach neural tissues and that the peptide is therefore unlikely to cross the blood brain barrier. This observation will limit the number of applications where vector delivery to the brain is required, with the potential exception of pathological conditions such as brain tumors in which disruption of the blood brain barrier is expected to occur. A high activity was detected in the stomach, spleen, skin, lungs, intestine, duodenum, pancreas and liver. Non negligible labeling was observed in the heart, skeletal muscle, thymus and brown fat. In some organs such as salivary glands and the thyroid, the accumulation of radioactivity was linked to specific accumulation of free ^125^I which could readily be inhibited by potassium perchlorate treatment [19].

Fourth, the strong renal and urine accumulation of radioactivity implies that the main route of elimination of the peptide was renal. Kidney labeling did not change significantly between 15 and 60 min, suggesting that rapid elimination occurred early following IV injection of the peptide. The only organs that demonstrated increased levels of radioactivity with time were those that accumulated free ^125^I suggesting that ^125^I-L-MCa distribution *in vivo* reached equilibrium rapidly following IV injection.

## 4. Materials and Methods

### 4.1. Materials

Lactoperoxidase from bovine milk, hydrogen peroxide 30% (*w*/*w*) in H_2_O were supplied from Sigma-Aldrich (St. Louis, MO, USA); Na^125^I (3.7 GBq/mL) from Perkin Elmer (Boston, BA, USA). Acetonitrile and trifluoroacetic acid were High Pressure Liquid Chromatography (HPLC) grade products and were purchased from Aldrich Chemical Co. (St, Louis, MO, USA). For radio-analyses: an HPLC apparatus (Shimadzu, Kyoto, Japan) equipped with NaI (Tl) scintillation detectors (LabLogic, Sheffield, UK) was used. Plasma and blood cell activities were assessed using a dose calibrator Capintec CRC-15R (Ramsey, NJ, USA). For *in vitro* stability centrifugate MiniSpin plus^®^ (Eppendorf, Hamburg, Garmany) and a 10 K Omega™ membrane (Nanosep^®^; Pall Life Science, East Hills, NY, USA) were used.

### 4.2. Chemical Synthesis and Radiolabeling

Chemical synthesis of Tyr-MCa and Lin-Tyr-MCa were performed as previously described [5,8]. Both MCa analogs were then radioiodinated according to Ahmadi *et al.* [15]. Briefly, 37 MBq of ^125^I were added to 10 µg of each peptide in phosphate buffer (50 mM, pH = 7.4). The reaction was allowed to proceed for 30 min at room temperature (RT) after addition of 8 µL (1 mg/mL) lactoperoxidase and 10 µL H_2_O_2_ (1:50,000).

Radioiodinated MCa analogues were then analysed by HPLC using a Vydac 218 TP C18 column, (10 µm, 4.6 × 250 mm). The solvent system consisted of H_2_O–TFA 0.1% (solvent A) and acetonitrile 90%–TFA 0.1% (solvent B). Tracer elution was achieved by applying a gradient of 0% B during 8.3 min, 0%–1% B during 1.66 min, 1%–10% B during 1.66 min, 10%–60% B during 33.2 min, 60%–100% B during 1.66 min, 100% B during 8.3 min and 100%–0% B during 8.3 min (total run time, 65 min) at a flow rate of 1 mL/min. The stability of the radiolabeling was determined by incubation of the radioiodinated complexes at RT for 24 h, following which, an HPLC analysis was performed as described above.

### 4.3. In Vitro Stability in Mouse Blood

The *in vitro* stability of ^125^I-Tyr-MCa and ^125^I-Lin-Tyr-MCa were evaluated in mouse blood. The experiments were performed in triplicate. Twenty MBq (5 µg) of each radiolabeled MCa analogue were incubated for 180 min in 0.5 mL of mouse blood at 37 °C, yielding a ^125^I-Tyr-MCa and ^125^I-Lin-Tyr-MCa concentration of 2.3 µM. At different time point between 15 min and 180 min, a 100 µL blood sample was removed and then centrifuged (2000× *g*, 5 min) (pellet, fraction #1). The separated plasma was filtered through a 10 K Omega™ membrane (Nanosep^®^; Pall Life Science, East Hills, NY, USA) at 7000× *g* for 20 min (filter, fraction #2; filtered solution, fraction #3) followed by HPLC analyses using the above mentioned conditions. The blood samples were also used for the determination of the relative distribution of radioactivity in fractions #1, #2, and #3 corresponding to blood cells, plasma proteins, and protein-free plasma, respectively. The potential presence of free ^125^I in fractions #1 and #2 was analyzed by TLC using RP18 as stationary phase and physiological serum as mobile phase. In this analytical system, free iodine migrates to the solvent front and radioiodinated peptides remain at the deposit point.

### 4.4. In Vivo Experimental Protocol

#### 4.4.1. Animals

Eight standard CD-1 mice were purchased from Charles River Laboratories (L’Arbresle, France) and housed for 1 week prior to inclusion in the experimental protocol. Conscious animals were restrained for tracer injection in a tail vein. All experimental procedures were performed in accordance with the European Ethics Committee on Animal Care Guidelines (Directive 86/609/EEC) and were approved by the local Animal Care and Use Committee of the University (ComEth Grenoble, C2EA n 12,2012).

#### 4.4.2. *In Vivo* ^125^I-MCa Stability

Two animals were dedicated to the evaluation of ^125^I-Tyr-MCa *in vivo* stability. The animals were pretreated by the NaI symporter inhibitor potassium perchlorate and then injected with 55 MBq (15 µg) of tracer (2.3 µM final *in vivo* concentration considering a total blood volume of 1.5 mL. At 15 and 30 min (*n* = 1) following injection, the animals were anesthetized using pentobarbital (60 mg/kg, IP) and a transmural puncture was performed in order to withdraw a blood sample directly from the left ventricular cavity. The blood sample was immediately centrifuged, the plasma was filtered, and the filtered solutions were analyzed by HPLC as described above. In addition, the distribution of radioactivity in fractions obtained as described above was also performed.

#### 4.4.3. Biodistribution

^125^I-Tyr-MCa (1.5 ± 0.0 MBq/g body weight; ~35 MBq total (10 µg, final *in vivo* concentration 1.5 µM considering a total blood volume of ~1.5 mL) was injected in one of the tail vein (IV) of 6 conscious and restrained animals (body weight, 23.7 ± 0.3 g) in the absence (*n* = 3) or presence (*n* = 3) of the NaI symporter inhibitor potassium perchlorate (3 mg/kg administered IV ~15 min prior to ^125^I-Tyr-MCa injection). Three out of 6 animals were anesthetized using isoflurane 1.5% (Aerrane, Baxter, France) in a 1:1 mixture of room air and oxygen, and then placed in a temperature-controlled bed for whole-body SPECT/CT acquisitions. SPECT/CT experiments were performed using a four-head gamma camera dedicated to small animals (NanoSPECT/CT, Bioscan/Mediso) equiped with 9-pinholes collimators of 1.4 diameter. The scans were acquired sequentially using Nucline software (Mediso, Budapest, Hungary) at 15, 30, and 60 min post-tracer injection. The SPECT parameters were: 24 projections and 20 sec per projection. The CT was performed between the 30 and 60 min nuclear image acquisition at 45 keV. Reconstruction of scans was carried out using HiSPECT NG (Bioscan, Goettingen, Germany). CT and SPECT acquisitions were reconstructed, fused and quantified using dedicated software (*InVivo* Scope, Bioscan). SPECT scale, obtained in kBq/voxel, was converted into percentage of injected dose per cm^3^ (%ID/cm^3^) and set to similar levels to allow direct visual comparison. Regions of interest (ROIs) were drawn on the thyroid, liver, kidney, skeletal muscle, stomach, salivary gland, brain, heart, myocardial left ventricle (for blood activity determination) and bladder.

#### 4.4.4. *Post Mortem* Analysis

Sixty min following tracer injection, all 6 anesthetized animals were euthanized by cervical dislocation and samples from the heart, lung, liver, spleen, kidney, stomach, intestine, duodenum, pancreas, salivary glands, thyroid, skin, skeletal muscle, pancreas, thymus, brain, fat, brown fat, spinal cord, blood, and urine were obtained. Radioactivity in organ and blood samples was measured thanks to a gamma-well counter (Cobra II, Packard Instruments, Courtaboeuf, France) with a 15–75 keV energy window for ^125^I. All tissue counts were corrected for background. Results were expressed as percentage of the injected dose per gram of wet weight (% ID/g).

### 4.5. Data Analysis

Data were given as mean ± SEM and compared using Student *t*-test and Kruskall–Wallis non parametric test (Systat software, San Jose, CA, USA) with a level of significance of 0.05.

## 5. Conclusions

In summary, the results from the present study indicated that MCa is a stable peptide vector *in vivo*, that it targets peripheral organs mainly with interesting quantitative differences, that blood cells also appear to accumulate the peptide and that the main route of elimination occurs through the kidneys. This study will therefore delineate the field of applications in which MCa could be used to deliver cargo into cells *in vivo*.
